# Inclusion of quantitative high-density plaque in coronary computed tomographic score system to predict the time of guidewire crossing chronic total occlusion

**DOI:** 10.1007/s00330-022-08564-2

**Published:** 2022-02-19

**Authors:** Rui Wang, Yi He, Haoran Xing, Dongfeng Zhang, Jinfan Tian, Yinghui Le, Lijun Zhang, Hui Chen, Xiantao Song, Zhenchang Wang

**Affiliations:** 1 Department of Radiology, Beijing Friendship Hospital, Capital Medical University, No. 95, Yong An Road, Xicheng District, Beijing, 100050 China; 2 Department of Radiology, Affiliated Hospital, Chengde Medical University, Chengde, Hebei China; 3 Department of Cardiology, Beijing Anzhen Hospital, Capital Medical University, Beijing, China; 4 Beijing Institute of Heart Lung and Blood Vessel Disease, Beijing, China; 5 Beijing Lab for Cardiovascular Precision Medicine, Beijing, China; 6 Department of Radiology, Beijing Anzhen Hospital, Capital Medical University, Beijing, China; 7 Department of Cardiology, Beijing Friendship Hospital, Capital Medical University, Beijing, China

**Keywords:** Coronary computed tomographic angiography, Predicting, Plaque quantitative analysis, Chronic total occlusion

## Abstract

**Objective:**

This study aimed to establish a new scoring system that includes histological quantitative features derived from coronary computed tomographic angiography (CCTA) to predict the efficiency of chronic total occlusion percutaneous coronary intervention (CTO-PCI).

**Methods:**

This study analyzed clinical, morphological, and histological characteristics of 207 CTO lesions in 201 patients (mean age 60.0 [52.0–65.0] years, 85% male), which were recruited from two centers. The primary endpoint was a guidewire successfully crossing the lesions within 30 m. The new predictive model was generated by factors that were determined by multivariate analysis. The CCTA plaque (CTAP) score that included a quantitative plaque characteristic was developed by assigning an appropriate integer score to each independent predictor, then summing all points. In addition, the CTAP score was compared with other predictive scores based on CCTA.

**Results:**

The endpoint was achieved in 63% of the lesions. The independent predictors included previous CTO-PCI failure, the proximal blunt stump, proximal side branch, distal side branch, occluded segment bending > 45°, and high-density plaque volume (fibrous volume + calcified volume) ≥ 19.9 mm^3^. As the score increased from 0 to 5, the success rate of the guidewire crossing within 30 m decreased from 96 to 0%. Comparing the CTAP score with other predictive scores, the CTAP score showed the highest discriminant power (c-statistic = 0.81 versus 0.73–0.77, *p* value 0.02–0.07). The CTAP score showed similar results for procedural success.

**Conclusion:**

The CTAP score efficiently predicted the guidewire crossing efficiency and procedural success.

**Key Points:**

• *An increase in high-density plaque volume (fibrous + dense calcium) was more probable to reduce the efficiency of crossing and lead to procedural failure.*

• *The new prediction scoring system with the addition of the quantitative characteristics of plaques had an improved predictive ability compared with the traditional prediction scoring system.*

**Supplementary Information:**

The online version contains supplementary material available at 10.1007/s00330-022-08564-2.

## Introduction

Coronary chronic total occlusion (CTO) is a common coronary arterial disease and is present in approximately 20% of the patients, which was revealed by coronary angiography (CAG) [1]. Percutaneous coronary intervention (PCI) is the mainstay of treatment of CTO (CTO-PCI). Successful CTO-PCI significantly reduces angina symptoms, improves left ventricular (LF) function, and increases long-term survival [2, 3]. However, CTO-PCI is technically more challenging than PCI for nonoccluded coronary diseases, and the failure of revascularization following CTO-PCI increases the risk of poor outcomes [4, 5]. Therefore, improving the success rate of CTO-PCI is particularly important for clinical benefits. Currently, coronary computed tomography angiography (CCTA) is the most important means of preoperative assessment of operation difficulty. Several existing predictive score systems, such as Computed Tomography Registry of Chronic Total Occlusion Revascularization (CT-RECTOR) score [6] and Korean Multicenter CTO CT Registry (KCCT) score [7], which were developed for CTO-PCI based on clinical factors and CCTA-measured morphological characteristics, exhibit appreciable predictive powers [8, 9].

Previous pathological studies showed that increased calcification and collagen fiber content in the CTO segment correlated well with the duration of the CTO-PCI treatment [10]. The increase in fibrous and calcified components resulted in denser and stiffer plaques, which might be the histological basis for the difficulty with a guidewire crossing the occluded segment. Recently, the development of plaque analysis software may perform quantitative analysis and accurately assess the histological characteristics of plaques [11, 12]. However, the current prediction model for CCTA is mainly based on the morphological characteristics of the lesion sites and the visually estimated severity of the calcified plaques; there is a lack of accurate qualitative and quantitative indicators of plaque histology in the CTO segment. Therefore, this study aimed to establish a new prediction scoring system based on clinical, morphological, and histological parameters that were determined by CCTA.

## Materials and methods

### Study design and subjection selection

This was a retrospective case-control study, and consecutively enrolled patients that underwent CCTA before CTO-PCI between September 2015 and September 2019 from two cardiac centers at Beijing Anzhen Hospital and Beijing Friendship Hospital, Beijing, China. CCTA was performed within 2 months before PCI. CTO was defined as complete occlusion of a coronary artery with a duration of > 3 months, and the thrombolysis in myocardial infarction (TIMI) grade was grade 0 in the occluded segment [13]. Patients that had one of the following were excluded from this study: a diameter of occluded vessels ˂ 2 mm; in-stent CTO; poor image quality for calcification or motion artifacts; and quantitatively uninterpretable lesions. The clinical baseline information for the patients was collected from the hospital’s medical record system. The institutional review board approval for this study was obtained at both centers (2020070X and 2020-P2-228-01). Due to the retrospective nature of our study, written informed consent was waived by the ethics committee of our hospital.

### Study endpoints

The primary endpoint was defined as a guidewire successfully crossing the CTO lesion within 30 m during the PCI procedure. This parameter was considered to objectively reflect the inherent level of procedural difficulty of a CTO lesion and to minimize the bias between the PCI outcome and the operator [14]. The secondary endpoint was defined as procedural success, for instance, the guidewire crossing the CTO segment successfully at any time, and the restoration of flow (residual stenosis < 30% and a TIMI flow of grade 3).

### CCTA protocol and image analysis

CCTA images were obtained in all patients by > 64-slice CT scanners (Somatom Definition FLASH, Siemens Healthcare; Revolution CT, General Electric; Aquilion ONE, Toshiba). Tube voltage was set at either 100 or 120 KV according to the body mass index (BMI) of the patient (100 KV for BMI < 24 kg/m^2^; 120 KV for BMI ≥ 24 kg/m^2^). The tube current was regulated by automatic exposure control. A contrast-enhanced scan was performed with 50–70 mL contrast media with a flow rate of 4–6 mL/s, followed by an injection of 30–50 mL saline into the tube. The phase with the least artifact in the cardiac cycle was selected and transmitted to the post-processing workstation.

CCTA image quality evaluation and plaque analysis were performed by two experienced imaging physicians who were blinded to the clinical data and coronary angiographic results. The images of the CTO segments were quantitatively analyzed using dedicated software (QAngioCT Research Edition Version v3.1.4 Medis). A semi-automatic or manual mode was used to extract the CTO segment path. The occlusive segment was defined as the coronary artery without contrast medium filling and was manually marked. The vessel contour of the occluded segment that was automatically identified by the software was routinely used unless it was identified incorrectly. Quantitative plaque measurements were performed independently within each occluded segment of the coronary artery. The software automatically acquired the length, total plaque volume, necrotic core volume, fibrofatty volume, fiber volume, calcification volume, and the remodeling index of the occluded segment. The thresholds for different plaque components were distinguished according to the CT values (Hounsfield units HU), which were necrotic nucleus (− 30 to 75 HU), fibrofatty (76–130 HU), fibrous tissues (131–350 HU), and calcification (> 351 HU) [15]. To facilitate clinical research and application, the fibrous component was defined combined with the calcified component as the high-density plaque component (> 130 HU). Quantitative data were automatically exported from the plaque analysis software and recorded.

Multiplanar reformation, volume rendering, and transaxial images were used to evaluate the morphological parameters of the occluded segment and its adjacent area. Each morphological parameter was independently observed and recorded. The proximal stump morphology was classified as tapered or blunt. The tortuosity of the occluded segment was defined as the presence of ≥ 1 bending > 45° throughout the entire occluded route. Multiple occlusion sites were defined as ≥ 2 complete interruptions of the contrast opacification separated by a contrast-enhanced segment of ≥ 5 mm [16]. The branches at the proximal and distal positions were defined as any visualized collateral branches within 3 mm near the tip and end of the occlusion [6]. Serious lesions adjacent to the proximal or distal position of the occlusive segment were defined as the vessel with a stenosis > 70% [7]. The assessment of calcification severity was performed as previously described in the KCCT and CT-RECTOR scores, respectively [6, 7], and the corresponding CTA-derived J-CTO score, KCCT score, and CT-RECTOR scores were calculated.

### Statistical analysis

Normally distributed continuous variables are expressed as mean ± standard deviation, otherwise as median (interquartile range). Categorical variables are expressed as frequencies (percentage). Univariate analysis was performed using the *t* test or Mann–Whitney *U* test for continuous variables, and Pearson’s chi-squared or Fisher’s exact test was used to examine the differences in categorical variables between groups.

Predictive models that showed that the guidewire crossed the lesion region successfully within 30 m were established by multivariate logistic regression analysis. Variables that were statistically significant in the univariate analysis were included in the multivariate analysis. To generate the scoring system, continuous variables with statistical significance in univariate analysis were analyzed by receiver operating curve (ROC) to obtain the optimal cutoff values under the endpoint event, which were then transformed into binary variables for multivariate analysis. Multivariate screening used a stepwise two-way entry approach. The final variables that resulted from the regression analysis were imported into the prediction model. The C-statistic of the new prediction model was calculated according to the method described by Delong et al [17], and the calibration curve of the model was plotted.

The new model was internally validated using the method of a 5-fold crossover test. The model development data were randomly partitioned into five equally sized subsets. Each time a subset was used as the validation dataset, the remaining four subsets were used as the model development dataset, and the cross-validation process was repeated five times. Each of the subsets was used once as the validation dataset. The mean C-statistic of the model after cross-validation was calculated, the calibration curve was plotted, and the Brier score was calculated.

The CTAP scoring system was established using the method of the Framingham Heart Study [18]. An appropriate integer score was assigned to each independent variable based on the model’s beta coefficients. The total score for each CTO was calculated by summing all points accrued. The discrimination and calibration of the new scoring system were compared with the previous scores based on CCTA.

All statistical significance was defined as two-tailed *p* < 0.05, and all statistical analyses were performed using R software (R version 4.0.2).

## Results

### Clinical characteristics

A total of 201 patients (mean age 60.0 [52.0, 65.0] years, 85% male) with 207 CTO lesions were included in this study. Among these 207 lesions, 63% were in the successful group, in which the guidewire crossed the lesions successfully within 30 m. However, most of the clinical characteristics of patients in the success group were similar to those of the failed group, except for a high proportion of previous failed CTO-PCI attempts(22.7% versus 7.9%, *p* = 0.006) (Table 1). Procedure success was achieved in 76% of lesions; however, the clinical characteristics of the patients in the successful group were similar to those in the failed group (Supplemental Table 1).

**Table 1:** Clinical characteristics between the guidewire crossing within 30 m for the successful group and failed group

Clinical characteristics	Total (*n* = 201)	Fail (*n* = 75)	Success (*n* = 126)	*p* value
Age (years)	60.00 (52.00, 65.00)	60.00 (52.00, 65.00)	60.00 (51.25, 65.00)	0.792
Gender (male)	170 (85)	66 (88)	104 (83)	0.404
BMI (kg/m)	26.27 ± 3.35	26.23 ± 3.58	26.30 ± 3.22	0.899
Occlusion duration ≥ 12 months or unknown	131 (65)	55 (73)	76 (60)	0.085
Hypertension	129 (64)	43 (57)	86 (68)	0.159
Diabetes mellitus	61 (30)	23 (31)	38 (30)	1
Hyperlipidemia	80 (40)	28 (37)	52 (41)	0.687
Smoking history	108 (54)	41 (55)	67 (53)	0.953
Heart failure	9 ( 4)	2 ( 3)	7 ( 6)	0.489
Renal disease	12 ( 6)	5 ( 7)	7 ( 6)	0.765
Previous CTO-PCI failure	27 (13)	17 (23)	10 ( 8)	0.006
Previous PCI	39 (19)	15 (20)	24 (19)	1
Previous CABG	2 ( 1)	2 ( 3)	0 ( 0)	0.138
Previous MI	51 (25)	16 (21)	35 (28)	0.396
Stroke	18 ( 9)	6 ( 8)	12 (10)	0.912
PVD	10 ( 5)	4 ( 5)	6 ( 5)	1
CAD family history	9 ( 4)	3 ( 4)	6 ( 5)	1

Per-patient analysis. Continuous variables are mean ± standard deviation (SD) or median (25th–75th percentile), and categorical variables are *n* (%)

*BMI* body mass index, *CTO-PCI* chronic total occlusion percutaneous coronary intervention, *PCI* percutaneous coronary intervention, *CABG* coronary artery bypass grafting, *MI* myocardial infarction, *PVD* peripheral vascular disease, *CAD* coronary heart disease

### Morphological and histological characteristics of CCTA

Univariate analysis showed that the characteristics that affected a guidewire crossing within 30 m included the left circumflex artery (LCX) lesions, the proximal blunt stump, proximal branches, distal branches, the occlusive segment bending > 45°, severe distal vascular lesions, lesion length, total plaque volume, fibrous fatty plaque volume, fibrous plaque volume, calcified plaque volume, and the high-density plaque volume, for instance, fibrous volume + calcified volume (Table 2). Similar results were obtained based on the secondary endpoint, except for distal branches and the lesions located in LCX (Supplemental Table 2).

**Table 2:** CCTA morphological and histological characteristics between the guidewire crossing ≤ 30 m successful group and failed group

	Total (*n* = 207)	Fail (*n* = 76)	Success (*n* = 131)	*p* value
Morphological characteristics				
Location of CTO lesion				
LAD	85 (41)	33 (43)	52 (40)	0.526
LCX	22 (11)	2 ( 3)	20 (15)	0.004
RCA	98 (47)	41 (54)	57 (44)	0.147
LM	2 ( 1)	0 ( 0)	2 ( 2)	0.533
Proximal blunt stump	74 (36)	43 (57)	31 (24)	0.001
Proximal branch	65 (31)	36 (47)	9 (22)	0.001
Distal branch	48 (23)	25 (33)	23 (18)	0.019
Bending > 45°	42 (20)	25 (33)	17 (13)	0.001
Multiple occlusion	10 ( 5)	4 ( 5)	6 ( 5)	1
Severe proximal disease	44 (21)	20 (26)	24 (18)	0.238
Severe distal disease	44 (21)	24 (32)	20 (15)	0.01
Vessel wall remodeling index	0.90 (0.64, 1.06)	0.92 (0.66, 1.05)	0.89 (0.64, 1.06)	0.799
Histological characteristics				
Lesion length (mm)	16.45 (9.54, 27.00)	24.78 (13.24, 34.65)	12.97 (7.23, 21.70)	< 0.001
Total plaque volume (mm^3^)	138.69 (64.62, 215.18)	186.56 (105.73, 301.45)	118.37 (50.05, 188.38)	< 0.001
Necrotic core volume (mm^3^)	61.03 (30.11, 113.04)	67.84 (36.81, 133.65)	59.73 (25.54, 103.65)	0.12
Fibrous fatty volume (mm^3^)	26.78 (12.55, 54.63)	36.98 (13.61, 66.56)	23.96 (10.16, 45.44)	0.006
Fibrous volume (mm^3^)	12.53 (3.18, 37.88)	25.64 (8.21, 65.36)	8.84 (2.05, 19.86)	< 0.001
Dense calcium volume (mm^3^)	0.12 (0.00, 7.06)	2.39 (0.00, 19.68)	0.00 (0.00, 3.37)	< 0.001
Fibrous + dense calcium volume (mm^3^)	15.06 (3.18, 45.37)	32.59 (9.19, 90.66	9.12 (2.05, 25.95)	< 0.001

Per-lesion analysis. Continuous variables are median (25th–75th percentile), and categorical variables are *n* (%)

*CTO* chronic total occlusion, *LM* left main coronary artery, *LAD* left anterior descending artery, *LCX* left circumflex, *RCA* right coronary artery, *D* diagonal branch

### Multivariate regression analysis and model development

Variables that were statistically significant in the univariate analysis were used in the multivariate analysis. Because of the severe collinearity between the total plaque volume and the lesion length that was revealed by Spearman’s correlation coefficient analysis (coefficient = 0.885), the total plaque volume was not included in the multivariate analysis. In addition, fibrous fatty components were considered as tissues that could not be clearly distinguished by the CT threshold in plaque analysis; therefore, fibrofatty plaques were not included in the multivariate analysis. The optimal cut-off values of the length and high-density plaque volume were 25.25 mm and 19.9 mm^3^, respectively. Both continuous variables were included in the multivariate regression analysis after binary transformation.

Therefore, one clinical variable, six CCTA morphologic variables, lesion length, and one CCTA plaque histological variable were included in the multivariate regression analysis. The results showed that previous CTO-PCI failure, the proximal blunt stump, proximal branch, distal branch, occlusive segment bending > 45°, and the high-density plaque volume (fibrous volume + calcified volume) ≥ 19.9 mm^3^ were independent predictors of guidewire crossing within 30 m. The final variables obtained from the multivariate analysis were imported into the prediction model. Table 3 shows the odds ratio and its 95% confidence interval (CI), *p* value, and the regression coefficient for each variable in the model. The C-statistic of the new model was 0.8181, and the ROC curve and the calibration curve of the model were plotted (Supplemental Figs. 1 and 2). The obtained calibration curve suggested good calibration of the new model.

**Table 3:** Logistics multivariate regression analysis

	Odds ratio (95% CI)	*p* value	*β* coefficient
Previous CTO-PCI failure	0.26 (0.10–0.69)	0.007	− 1.34
Proximal blunt stump	0.43 (0.21–0.90)	0.024	− 0.84
Proximal branch	0.35 (0.16–0.75)	0.007	− 1.04
Distal branch	0.39 (0.17–0.86)	0.02	− 0.94
Bending > 45°	0.29 (0.12–0.66)	0.003	− 1.24
High-density plaque volume ≥ 19.9 mm^3^	0.28 (0.14–0.55)	< 0.001	− 1.29

### Internal validation of the model

The mean C-statistic of the 5-fold cross-validation was 0.8003, which was similar to the original model. The Brier score was 0.17600, and the calibration curve showed good cross-validation calibration (Supplemental Fig. 3). Therefore, the fitting process of the original model was considered to be reproducible.

### Development of the CTAP score

**Fig. 1:**
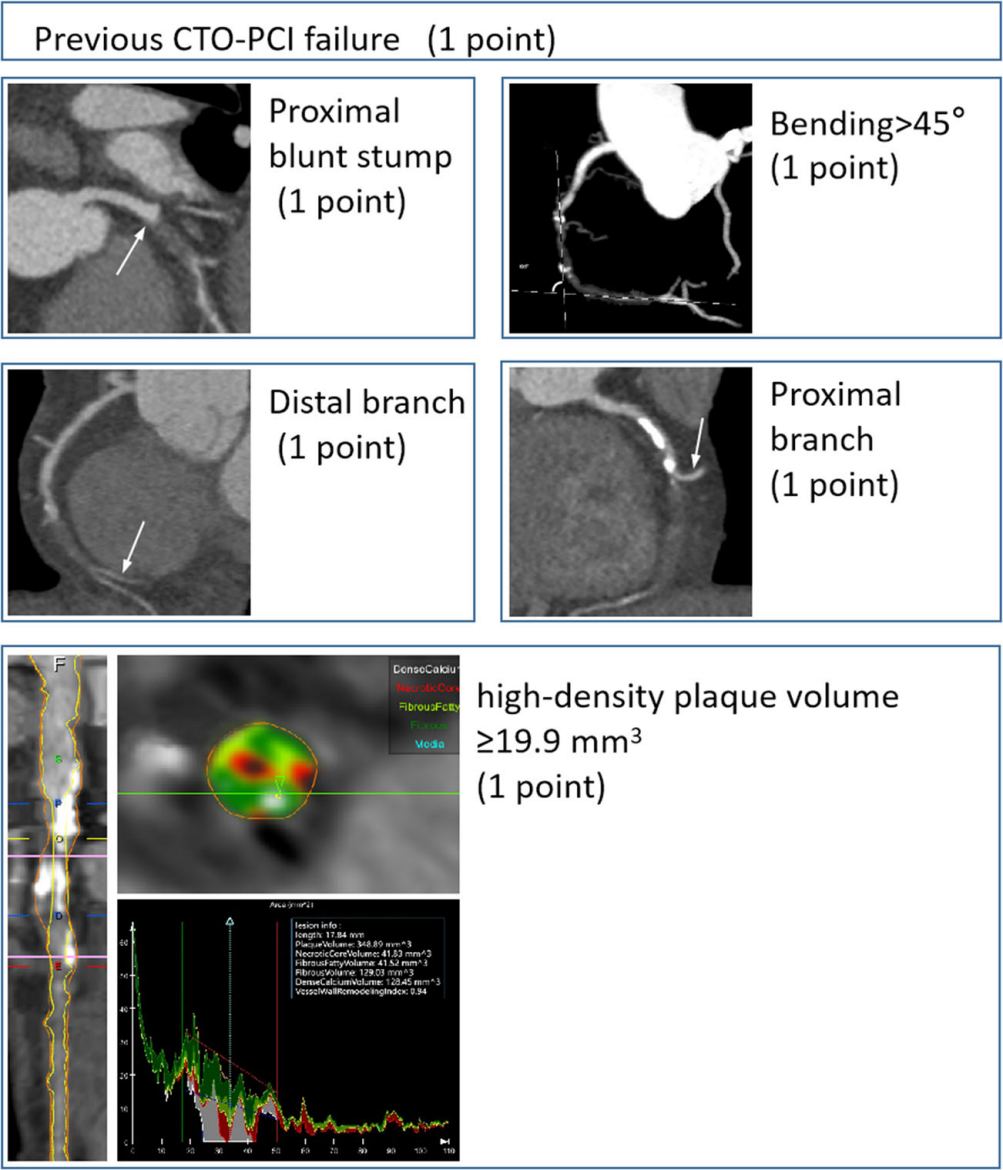
Summary of CTAP score

**Fig. 2:**
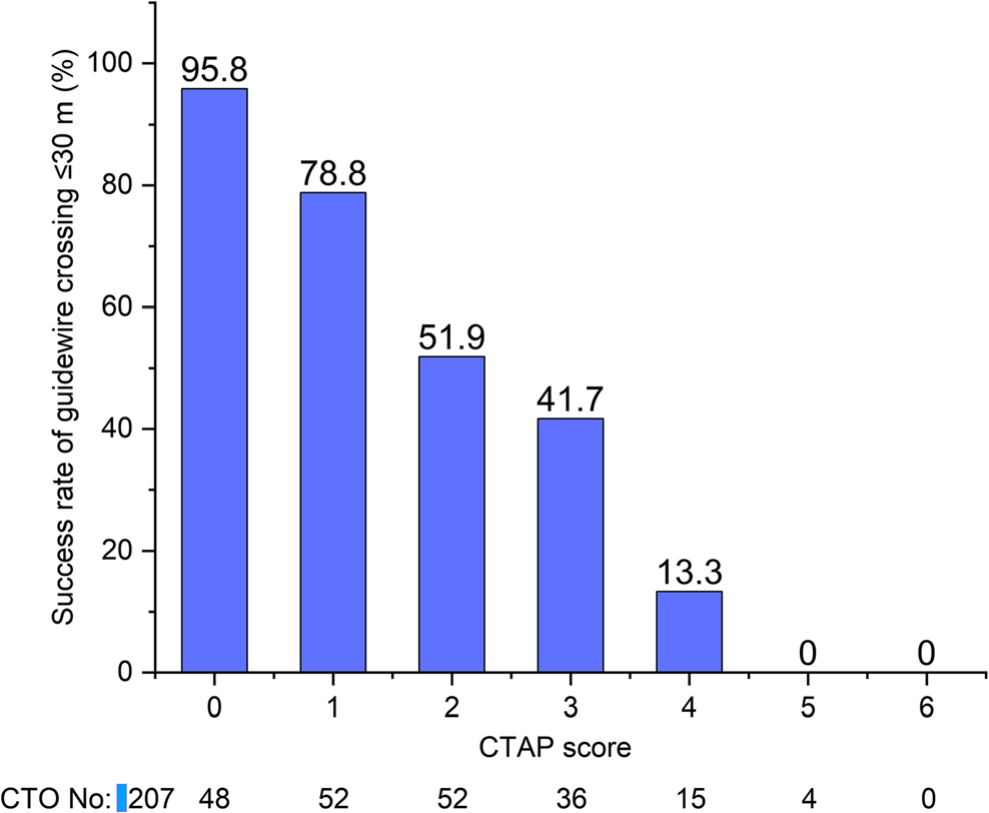
Relationship between the CTAP score and the success rate of guidewire crossing within 30 m

Based on the regression model’s beta coefficients that were from 0.84 to 1.34, an integer of one was calculated and finally assigned to each independent variable and scored one. For each CTO lesion, a total CTAP score was calculated by summing all points accrued (Fig. 1). The relationship between the CTAP score and the success rate of guidewire crossing within 30 m decreased from 96 to 0% as the score gradually increased from 0 to 5 (Fig. 2). The procedure success rate gradually decreased with the increase in the CTAP score (Supplemental Fig. 4).

### Evaluation of the new scoring system

**Fig. 3:**
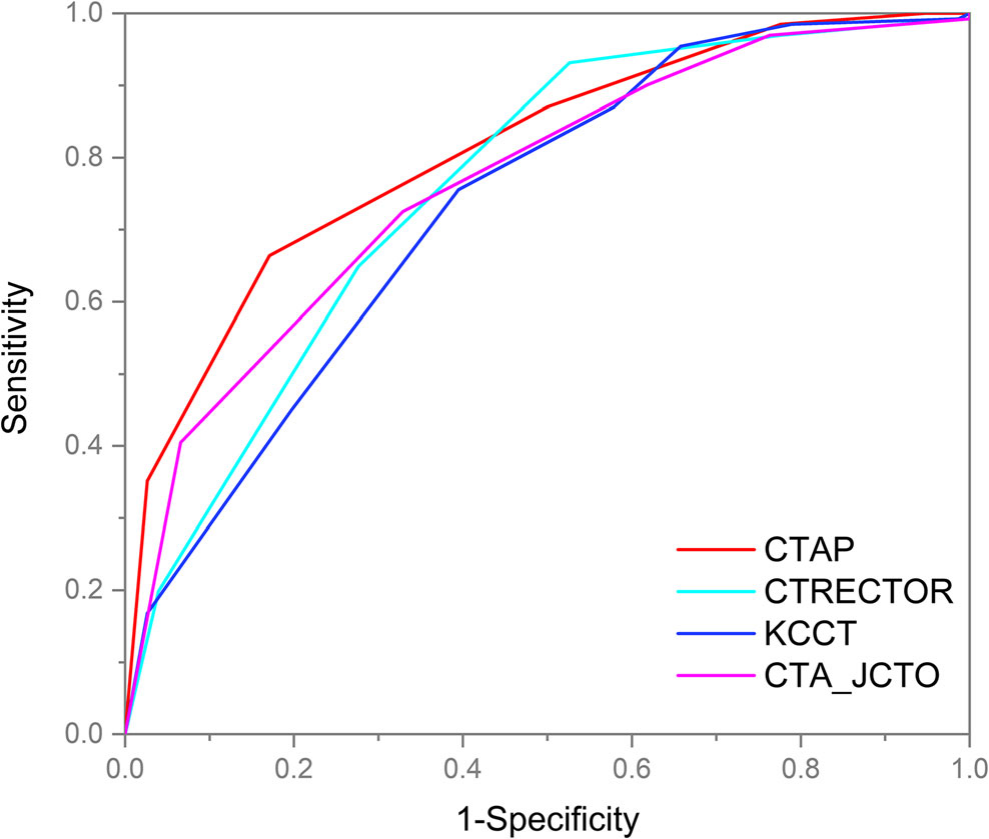
Comparison of the predictive performance of different scoring systems for guidewire crossing within 30 m

The CTAP score showed a good predictive ability for guidewire crossing within 30 m. The area under the ROC curve was 0.809. Compared with other CCTA-based scoring systems, the CTAP scores showed the highest discriminant ability (Fig. 3). The C-statistic of the CTAP score was statistically significant compared with KCCT (Table 4). In addition, this CTAP score system was used to predict the procedure success. This new system showed the best discriminant ability compared with other scoring systems (Supplemental Fig 5), although the C-statistic was 0.774 and not statistically different from other scoring systems (Supplemental Table 3).

**Table 4:** Comparison of C-statistics between the CTAP score and other scoring systems for guidewire crossing within 30 m

Variable	AUC	SE^a^	95% CI^b^	*p* value
CTAP	0.809	0.0291	0.749 to 0.860	
CTA-derived J-CTO	0.765	0.0325	0.701 to 0.821	0.0678
KCCT	0.732	0.0360	0.666 to 0.791	0.0187
CT-RECTOR	0.759	0.0342	0.695 to 0.816	0.0786

^a^DeLong et al (1988)

^b^Binomial exact

## Discussion

In this retrospective study, the relationship between qualitative and quantitative histological features of CTO lesions with the outcomes of CTO-PCI was investigated, which showed that an increase in high-density plaque volume (fibrous volume + calcified volume) was more probable to reduce the crossing efficiency and lead to procedural failure. A CTAP score system was generated that used multivariate analysis to include the high-density plaque volume into the traditional CCTA morphological parameters and clinical parameters. A high-density plaque volume ≥ 19.9 mm^3^ remained an independent predictor of guidewire crossing within 30 m, and that the new CTAP prediction scoring system with the addition of quantitative characteristics of plaques had a better predictive ability than traditional prediction scoring systems.

Understanding the histological characteristics of CTO lesions is a prerequisite for successful CTO-PCI procedures [19]. Pathological studies confirmed that CTO lesions of a short duration presented with a soft thrombus and necrotic core, whereas CTO lesions of a long duration had predominantly firm fibrous and calcified tissue components [20–22]. Several CTA studies of plaque progression showed that the density of noncalcified components in advanced occlusive lesions was significantly higher than that in the early stages and that the advanced CTO lesions had more severe calcification than those at an early stage [23, 24]. This study showed that the total plaque volume and the high-density plaque volume significantly increased in the failed procedure group, for instance, the group where guidewire crossing took > 30 m, and this finding could be a valid supplement to previous studies. In addition, the results from this study showed that the effects of low-density plaque volume on procedural efficiency and outcomes were not statistically significant, which suggested that more attention should be given to the quantity of high-density plaques before performing CTO-PCI.

Currently, CAG or CCTA-based scoring systems are widely used to assess the difficulty of CTO during PCI. Common morphological predictors include the length of a lesion, bending of the occluded segment, proximal stump morphology, multiple occlusion, proximal branch, and severe calcification. Clinical predictors include the duration of CTO ≥ 12 months, failure of the previous PCI, previous myocardial infarction, and a history of coronary artery bypass grafting [6, 7, 14, 25–27]. The predictive variables in each scoring system vary, and significant differences in prediction performance between different scoring systems exist. Among these predictive variables, severe calcification was confirmed to be the main obstacle to CTO-PCI by most scoring systems [6, 7, 14, 25, 27]. However, the current CCTA-based scoring system evaluates the degree of calcification by subjectively identifying the location of calcification, and therefore, there is a lack of objective quantitative indicators [6, 7, 9]. In this study, during the assessment of calcification, the location of calcification was often difficult to judge due to the effect of the calcification pseudo-shadow. In contrast, previous scoring systems did not consider the effect of fibrous plaques on CTO-PCI. Therefore, the calcified and fibrous plaques were defined as high-density plaques, which showed that the high-density plaques were independent factors that affected the efficiency of guidewire crossing. This finding further confirmed the histological features of advanced CTO in pathological studies and improved the understanding of the effect of plaque composition on the outcomes of CTO-PCI.

In most studies, the length of an occluded segment was considered as an independent factor that affected the outcomes of PCI [7, 14, 25, 27]. In this study, the length of the occluded segment was statistically significant in the univariate analysis but did not remain in the model following multivariate regression analysis, which might be because that the high-density plaque volume was introduced into the multivariate analysis that affected the procedural outcomes more significantly. For length, the high-density plaque volume reflected the amount of hard tissue in the CTO segment; therefore, this was more important for the procedural outcome. Bending of the CTO segments and proximal blunt stump have often been reported as risk factors for procedural outcomes, which agreed with the findings from this study. The univariate analysis of the site of CTO lesions showed that LCX lesions were different and that the other sites of disease were not statistically significant. However, in contrast to a previous study [26], the guidewire tended to cross the LCX lesions more easily, which might be explained by the heterogeneous data of this group. In addition, the new prediction model showed that the proximal and distal branches were independent factors for CTO-PCI efficiency. The proximal branch was reported as an independent factor in the KCCT score [7]; however, the distal branch was not in the known scoring models. The univariate analysis showed that the distal branch was statistically significant between the success and failed group, but did not show any statistical significance between the success and failure of the procedure. Therefore, the presence of the distal branch might affect the direction during the guidewire marching process, and therefore, the guidewire crossing efficiency, but had a limited effect on the final success of the procedure.

In this study, recruited patients were recruited form the last 5 years, which reflected the current status of the difficulties with CTO-PCI. Compared with the previous scoring systems that were developed based on CCTA, the new scoring system that included plaque components exhibited the highest discriminant ability. The inclusion of quantitative indicators of high-density plaques in a scoring system made the predictor more objective without increasing the number of predictors, which significantly improved the ability to predict the guidewire crossing efficiency, and had a predictive value for the procedural outcomes, for example, success or failure. The new system could promote the application of intelligent plaque analysis software in clinical practice.

Some limitations of this study should be acknowledged. First, this was a retrospective observational study and, therefore, potentially had case selection bias. Second, because only consecutively recruited CTO patients for the past nearly 5 years were included, the sample size was insufficient to extrapolate the model into broader applications. Therefore, further assessment of the applicability of this new model in a large cohort population is required. Third, in this study, reverse patency was only attempted on two patients. Therefore, a stratified study of reverse patency was not carried out.

## Conclusion

The prediction model that included the high-density plaque volume improved the predictive ability, which effectively predicted the guidewire crossing efficiency and predicted the procedural outcomes, which were success or failure.

## Supplementary Information

ESM 1 (DOCX 429 kb)

## Declarations

### Guarantor

The scientific guarantor of this publication is Zhenchang Wang.

### Statistics and biometry

No complex statistical methods were necessary for this paper.

### Methodology

• retrospective

## Abbreviations

BMI: Body mass index
CABG: Coronary artery bypass grafting
CAD: Coronary artery disease
CAG: Coronary angiography
CCTA: Coronary computed tomographic angiography
CTA-derived score: J-CTO score derived from computed tomography angiography
CTO: Chronic total occlusion
CT-RECTOR: Computed Tomography Registry of Chronic Total Occlusion Revascularization
KCCT: Korean Multicenter CTO CT Registry
PCI: Percutaneous coronary intervention
TIMI: Thrombolysis in myocardial infarction

## References

[1] Fefer P, Knudtson ML, Cheema AN (2012). Current perspectives on coronary chronic total occlusions: the Canadian Multicenter Chronic Total Occlusions Registry. J Am Coll Cardiol.

[2] Tian J, Zuo H, Zhang L (2020). The success of opening concurrent chronic total occlusion lesion to improve cardiac function trial in patients with multi-vessel disease (SOS-moral): study protocol of a prospective multicenter study. Medicine (Baltimore).

[3] Roth C, Goliasch G, Aschauer S (2020). Impact of treatment strategies on long-term outcome of CTO patients. Eur J Intern Med.

[4] Bijuklic K, Schwencke C, Schofer J (2017). Long-term major adverse cardiac and cerebrovascular events (MACCE) rate: comparison of retrograde and antegrade recanalization of chronic total coronary occlusions. Wien Klin Wochenschr.

[5] Parsh J, Seth M, Green J (2017). Coronary artery perforations after contemporary percutaneous coronary interventions: evaluation of incidence, risk factors, outcomes, and predictors of mortality. Catheter Cardiovasc Interv.

[6] Opolski MP, Achenbach S, Schuhbäck A (2015). Coronary computed tomographic prediction rule for time-efficient guidewire crossing through chronic total occlusion: insights from the CT-RECTOR multicenter registry (Computed Tomography Registry of Chronic Total Occlusion Revascularization). JACC Cardiovasc Interv.

[7] Yu CW, Lee HJ, Suh J et al (2017) Coronary computed tomography angiography predicts guidewire crossing and success of percutaneous intervention for chronic total occlusion: Korean multicenter cto ct registry score as a tool for assessing difficulty in chronic total occlusion percutaneous coronary intervention. Circ Cardiovasc Imaging 10:e005800 10.1161/CIRCIMAGING.116.005800 28389507

[8] Tan Y, Zhou J, Zhang W (2017). Comparison of CT-RECTOR and J-CTO scores to predict chronic total occlusion difficulty for percutaneous coronary intervention. Int J Cardiol.

[9] Fujino A, Otsuji S, Hasegawa K (2018). Accuracy of J-CTO score derived from computed tomography versus angiography to predict successful percutaneous coronary intervention. JACC Cardiovasc Imaging.

[10] Srivatsa SS, Edwards WD, Boos CM (1997). Histologic correlates of angiographic chronic total coronary artery occlusions: influence of occlusion duration on neovascular channel patterns and intimal plaque composition. J Am Coll Cardiol.

[11] van Rosendael AR, Narula J, Lin FY (2020). Association of high-density calcified 1k plaque with risk of acute coronary syndrome. JAMA Cardiol.

[12] Smit JM, van Rosendael AR, El Mahdiui M (2020). Impact of clinical characteristics and statins on coronary plaque progression by serial computed tomography angiography. Circ Cardiovasc Imaging.

[13] Galassi AR, Werner GS, Boukhris M (2019). Percutaneous recanalisation of chronic total occlusions: 2019 consensus document from the EuroCTO Club. EuroIntervention.

[14] Morino Y, Abe M, Morimoto T (2011). Predicting successful guidewire crossing through chronic total occlusion of native coronary lesions within 30 minutes: the J-CTO (Multicenter CTO Registry in Japan) score as a difficulty grading and time assessment tool. JACC Cardiovasc Interv.

[15] Kim U, Leipsic JA, Sellers SL (2018). Natural history of diabetic coronary atherosclerosis by quantitative measurement of serial coronary computed tomographic angiography: results of the paradigm study. JACC Cardiovasc Imaging.

[16] Opolski MP, Kepka C, Achenbach S (2012). Coronary computed tomographic angiography for prediction of procedural and intermediate outcome of bypass grafting to left anterior descending artery occlusion with failed visualization on conventional angiography. Am J Cardiol.

[17] DeLong ER, DeLong DM, Clarke-Pearson DL (1988). Comparing the areas under two or more correlated receiver operating characteristic curves: a nonparametric approach. Biometrics.

[18] Sullivan LM, Massaro JM, D'Agostino RB (2004). Presentation of multivariate data for clinical use: The Framingham Study risk score functions. Stat Med.

[19] Mishra S (2017). Unraveling the mystique of CTO interventions: tips and techniques of using hardware to achieve success. Indian Heart J.

[20] Tran P, Phan H, Shah SR, Latif F, Nguyen T (2015). Applied pathology for interventions of coronary chronic total occlusion. Curr Cardiol Rev.

[21] Katsuragawa M, Fujiwara H, Miyamae M, Sasayama S (1993). Histologic studies in percutaneous transluminal coronary angioplasty for chronic total occlusion: comparison of tapering and abrupt types of occlusion and short and long occluded segments. J Am Coll Cardiol.

[22] Sakakura K, Nakano M, Otsuka F (2014). Comparison of pathology of chronic total occlusion with and without coronary artery bypass graft. Eur Heart J.

[23] Wu Q, Yu M, Li Y (2018). Natural history of untreated coronary total occlusions revealed with follow-up semi-automated quantitative coronary ct angiography: the morphological characteristics of initial ct predict occlusion shortening. Korean J Radiol.

[24] Yu M, Xu N, Zhang J (2015). CT features in the early and late stages of chronic total coronary occlusions. J Cardiovasc Comput Tomogr.

[25] Szijgyarto Z, Rampat R, Werner GS (2019). Derivation and validation of a chronic total coronary occlusion intervention procedural success score from the 20,000-patient eurocto registry: the eurocto (castle) score. JACC Cardiovasc Interv.

[26] Christopoulos G, Kandzari DE, Yeh RW (2016). Development and validation of a novel scoring system for predicting technical success of chronic total occlusion percutaneous coronary interventions: the progress cto (prospective global registry for the study of chronic total occlusion intervention) score. JACC Cardiovasc Interv.

[27] Alessandrino G, Chevalier B, Lefèvre T (2015). A clinical and angiographic scoring system to predict the probability of successful first-attempt percutaneous coronary intervention in patients with total chronic coronary occlusion. JACC Cardiovasc Interv.

