# Supplementary material for: Inclusion of quantitative high-density plaque in coronary computed tomographic score system to predict the time of guidewire crossing chronic total occlusion
Source: Eur Radiol. 2022 Feb 19;32(7):4565–73. doi: 10.1007/s00330-022-08564-2 (PMC9213281; doi:10.1007/s00330-022-08564-2)
Supplement: Supplementary file 1 — (DOCX 429 kb) [file 330_2022_8564_MOESM1_ESM.docx]

**Supplement Figure Legends**

**Supplement Figure 1**. ROC curves of the new model for successful guidewire crossing within 30


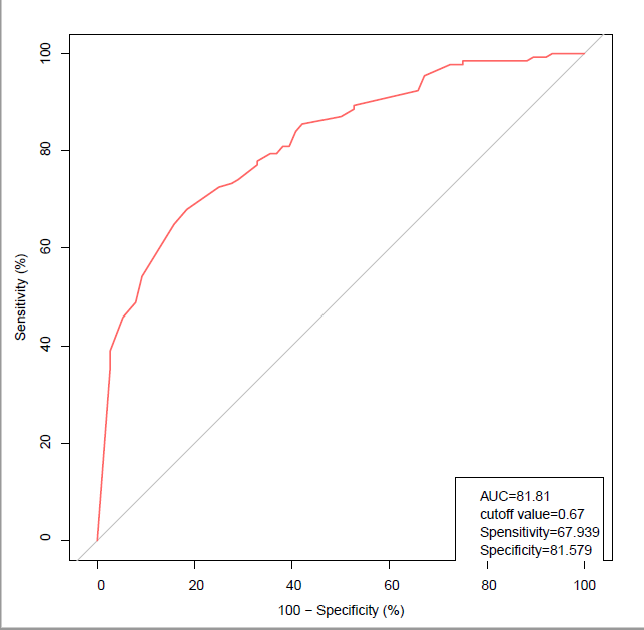


**Supplement Figure 2. Calibration curve of the new model**


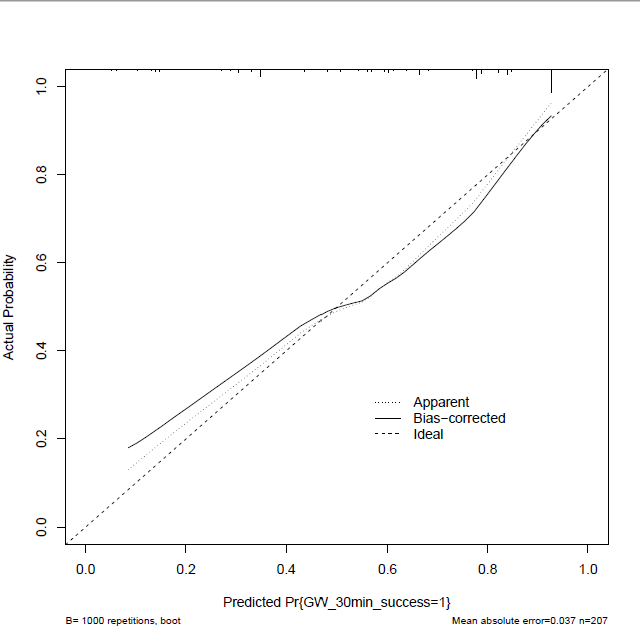


**Supplement Figure 3.** Calibration curve of the new model cross-validation


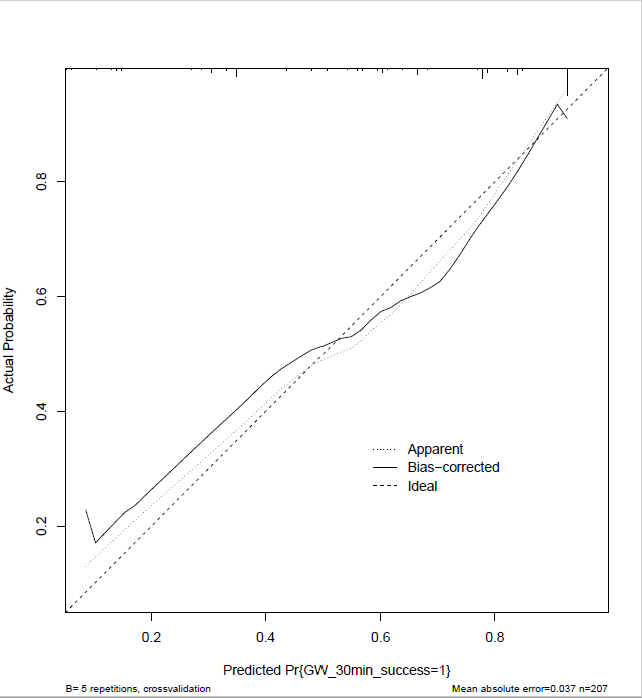


**Supplement Figure 4.** Relationship between CTAP score and the success rate of procedure


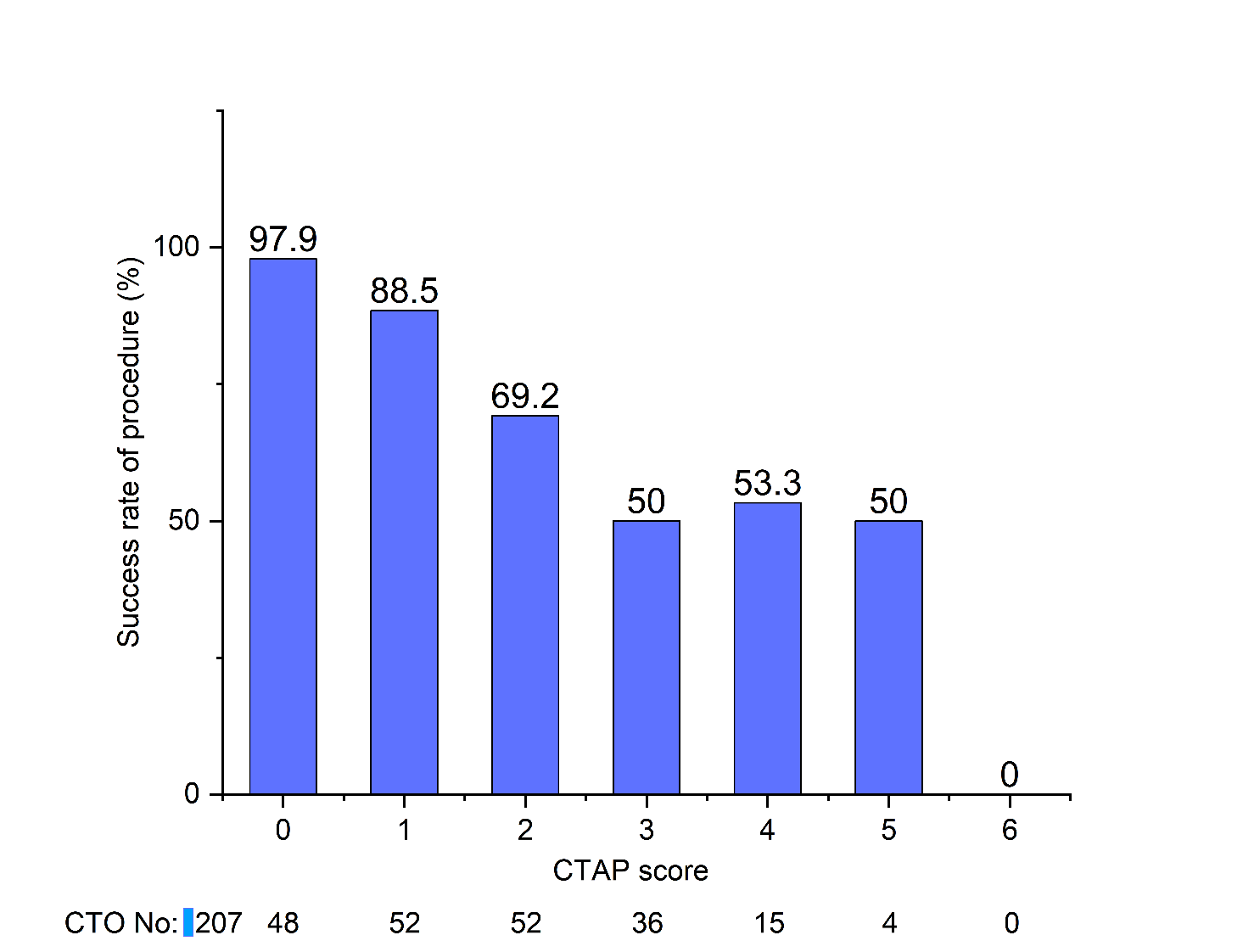


**Supplement Figure 5.** Comparison of predictive performance of different scoring systems for procedural success


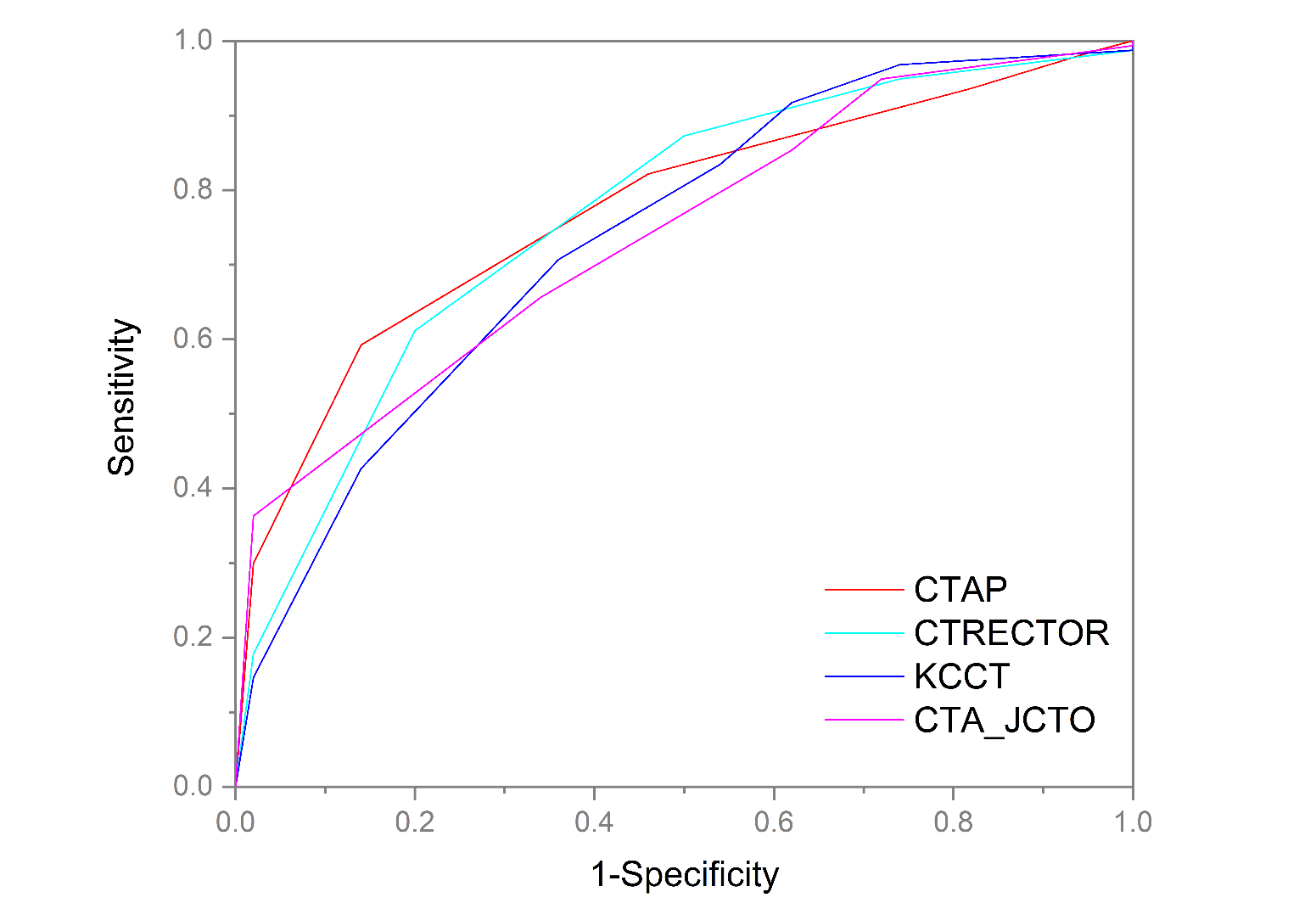


Supplemental Table 1. Clinical characteristics between the procedure successful group and failed group

| Variables | Total (n = 201) | Fail (n = 49) | Success (n = 152) | *p-*value |
| --- | --- | --- | --- | --- |
| Age (years) | 60.00 (52.00, 65.00) | 61.00 (57.00, 66.00) | 60.00 (50.75, 64.00) | 0.137 |
| Gender (male) | 170 (85) | 42 (86) | 128 (84) | 0.979 |
| Weight (kg) | 76.20 ± 13.67 | 73.36 ± 12.43 | 77.11 ± 13.97 | 0.082 |
| Height (cm) | 170.00 (165.00, 175.00) | 170.00 (163.00, 174.25) | 170.00 (165.00, 175.00) | 0.313 |
| BMI (kg/m) | 26.27 ± 3.35 | 25.76 ± 3.42 | 26.44 ± 3.32 | 0.229 |
| Occlusion duration ≥12months or unknow | 131 (65) | 38 (78) | 93 (61) | 0.055 |
| Hypertension | 129 (64) | 26 (53) | 103 (68) | 0.09 |
| DM | 61 (30) | 12 (24) | 49 (32) | 0.397 |
| Hyperlipidemia | 80 (40) | 18 (37) | 62 (41) | 0.737 |
| Smoking history | 108 (54) | 28 (57) | 80 (53) | 0.699 |
| Heart failure | 9 (4) | 1 (2) | 8 (5) | 0.691 |
| Renal disease | 12 (6) | 3 (6) | 9 (6) | 1 |
| Previous CTO-PCI failure | 27 (13) | 9 (18) | 18 (12) | 0.356 |
| Previous PCI | 39 (19) | 10 (20) | 29 (19) | 1 |
| Previous CABG | 2 (1) | 2 (4) | 0 (0) | 0.059 |
| Previsou MI | 51 (25) | 14 (29) | 37 (24) | 0.687 |
| Stroke | 18 (9) | 3 (6) | 15 (10) | 0.57 |
| PVD | 10 (5) | 3 (6) | 7 (5) | 0.708 |
| CAD family history | 9 (4) | 0 (0) | 9 (6) | 0.117 |

Per-patient analysis. Continuous variables are mean ± standard deviation (SD) or median (25th–75th percentile), and Categorical variables are n (%).

BMI = body mass index; CTO-PCI = chronic total occlusion percutaneous coronary intervention; PCI = percutaneous coronary intervention; CABG = coronary artery bypass grafting; MI= myocardial infarction; PVD= peripheral vascular disease; CAD= coronary heart disease.

Supplemental Table 2. CCTA morphological and histological characteristics between the procedure successful group and failed group

| Variables | Total (n = 207) | fail (n = 50) | success (n = 157) | *p-*value |
| --- | --- | --- | --- | --- |
| **Morphological characteristics** |  |  |  |  |
| Location of CTO lesion |  |  |  | 0.107 |
| LAD | 85 (41) | 21 (42) | 64 (40) |  |
| LCX | 22 (11) | 1 (2) | 21 (13) |  |
| RCA | 98 (47) | 28 (56) | 70 (45) |  |
| LM | 2 (1) | 0 (0) | 2 (1) |  |
| Proximal blunt stump | 74 (36) | 31 (62) | 43 (27) | <0.001 |
| Proximal branch | 65 (31) | 22 (44) | 43 (27) | 0.042 |
| Distal branch | 48 (23) | 14(28) | 34 (22) | 0.463 |
| Bending >45° | 42 (20) | 17 (34) | 25 (16) | 0.010 |
| Multiple occlusion | 10 (5) | 2 (4) | 8 (5) | 1 |
| Severe proximal disease | 44 (21) | 13 (26) | 31 (20) | 0.457 |
| Severe distal disease | 44 (21) | 18 (36) | 26 (17) | 0.006 |
| Vessel wall remodeling index | 0.90 (0.64, 1.06) | 0.88 (0.68, 1.06) | 0.90 (0.64, 1.05) | 0.681 |
| **histological characteristics** |  |  |  |  |
| Lesion length (mm) | 16.45 (9.54, 27.00) | 24.78 (13.62, 35.35) | 13.85 (7.93, 23.75) | <0.001 |
| Plaque volume (mm^3^) | 138.69 (64.62, 215.18) | 181.99(126.11,325.48) | 126.82(52.90, 196.13) | <0.001 |
| Necrotic core volume (mm^3^) | 61.03 (30.11, 113.04) | 68.30 (38.61, 127.86) | 59.73 (26.71, 105.51) | 0.244 |
| Fibrous fatty volume (mm^3^) | 26.78 (12.55, 54.63) | 33.78 (17.67, 69.63) | 24.25 (11.24, 52.39) | 0.019 |
| Fibrous volume (mm^3^) | 12.53 (3.18, 37.88) | 28.48 (14.48, 66.04) | 9.12 (2.57, 25.06) | <0.001 |
| Dense calcium volume (mm^3^) | 0.12 (0.00, 7.06) | 3.60 (0.11, 33.00) | 0.00 (0.00, 4.97) | <0.001 |
| Fibrous + dense calcium volume (mm^3^) | 15.06 (3.18, 45.37) | 39.18 (17.78, 96.97) | 10.26 (2.57, 33.67) | < 0.001 |

Per-lesion analysis. Continuous variables are median (25th–75th percentile), and categorical variables are n (%).

CTO = chronic total occlusion; LM= left main coronary artery; LAD = left anterior descending artery; LCX = left circumflex; RCA = right coronary artery; D=diagonal branch.

Supplemental Table 3. Comparison of C-statistics between CTAP score and other scoring system for procedure success

| Variable | AUC | SE ^a^ | 95% CI ^b^ | *p-*value |
| --- | --- | --- | --- | --- |
| CTAP | 0.774 | 0.0336 | 0.710 to 0.829 |  |
| CTA JCTO | 0.740 | 0.0358 | 0.675 to 0.798 | 0.2060 |
| KCCT | 0.737 | 0.0400 | 0.671 to 0.795 | 0.3176 |
| CT-RECTOR | 0.766 | 0.0369 | 0.702 to 0.822 | 0.7971 |

^a^ _DeLong et al., 1988_

^b^ _Binomial exact_
